# Value-based health care in mobile integrated health for acute elderly care: A qualitative study of health care professionals in Finland

**DOI:** 10.1097/HMR.0000000000000483

**Published:** 2026-07-02

**Authors:** Fan Wang, Sanna Pekanoja, Petri Ahokangas, Miia Jansson

**Affiliations:** Fan Wang, PhD, is a postdoctoral researcher at Martti Ahtisaari Institute, Oulu Business School, and Research Unit of Health Sciences and Technology, University of Oulu, Oulu, Finland. E-mail: fan.wang@oulu.fi.; Sanna Pekanoja, MHSc, is Doctoral Researcher, Research Unit of Health Sciences and Technology, University of Oulu, Oulu, Finland, and Emergency Department, Oulu University Hospital, Oulu, Finland.; Petri Ahokangas, PhD, is Professor, Martti Ahtisaari Institute, Oulu Business School, University of Oulu, Oulu, Finland; Miia Jansson, PhD, RN, is Professor, Research Unit of Health Sciences and Technology, University of Oulu, Oulu, Finland, Medical Research Center Oulu, Oulu University Hospital and University of Oulu, Oulu, Finland, and RMIT University, Melbourne, Australia

**Keywords:** value-based health care, mobile integrated health, value creation, delivery and capture, elderly care

## Abstract

**Background:**

Health care service providers face increasing challenges in delivering high-quality care due to an aging population, workforce shortages, and limited financial budgets. Mobile Integrated Healthcare (MIH) offers an alternative value-based solution for elderly patients with manageable acute conditions at home. Finland is piloting this model, but its value, task redesign, and cost-efficiency require thorough evaluation before formal integration.

**Purpose:**

This study explores the value of MIH for patients and the health care system through the lens of value creation, delivery, and capture.

**Methods:**

Qualitative data were collected via semistructured interviews with 21 frontline health care professionals (HCPs) involved in Finland’s MIH service.

**Results:**

MIH provides human-centered acute care for the elderly, enabling convenient access to emergency services at home and reducing unnecessary hospital visits. Value is cocreated through integrated networks of emergency and social services, leveraging paramedics’ and geriatric nurses’ expertise while standardizing care pathways. Effective implementation requires coordination and task-shifting across emergency departments, MIH teams, and social care providers.

**Conclusions:**

MIH enhances care quality, supports elderly independence, and contributes to the sustainability of the health care system by reducing emergency interventions and hospitalizations.

**Practical Implications:**

Health managers should prioritize skill development for health care professionals (HCPs), integration across governance, service, HCPs, and patient levels, and the establishment of coordinated information systems. This study offers policymakers a valuable example of how MIH can be organized within a collective, publicly funded health care system to promote equity, accessibility, and sustainability for value-based health care.

Patients aged 65 and older frequently use emergency services (Louras et al., 2023). A study in Finland revealed that one in five older adults leaves the emergency department (ED) without a specific diagnosis (Ukkonen et al., 2019). Similarly, the Australian Institute of Health and Welfare reported that in 2021–2022, more than 3 million Australians visited emergency departments for conditions that could have been managed in primary care AIHW (Australian Institute of Health and Welfare 2024). This reflects a broader global challenge, as many countries face similar pressures on emergency services due to limited access to timely and effective primary care, highlighting the need for a new emergency care model to reduce unnecessary visits.

Aging has become a universal issue affecting both developed and developing countries, such as the United States, China, Australia, and those across Europe, with the older population forming the largest group of health care users. This trend places significant pressure on EDs and health care systems. Shifting responsibilities to the community level may help address the social challenges of aging. In this context, Mobile Integrated Healthcare (MIH) offers a promising model for delivering proactive, community-based services tailored to the complex needs of older patients.

MIH delivers care directly to frequent ED users’ homes for nonemergent or emergent/primary care treatable conditions (Nejtek et al., 2017). After the introduction of MIH, there has been a significant reduction in transportation to ED (Lurie et al., 2023; O’Connor et al., 2023), resulting in substantial cost savings and enhanced resource utilization based on a statistical analysis of the ED cohort and its associated costs (Gingold et al., 2021; Xie et al., 2021). The MIH model improves health outcomes, experiences, and resource efficiency (Hughes et al., 2020).

Unlike the traditional hospital-centered emergency care model, which requires patients to travel to hospitals, the MIH provides a more human-centric and value-based approach by addressing acute illnesses directly in patients’ homes (Frazer, 2022). MIH is a community-based approach aligned with the Integrated People-Centered Health Services (IPCHS) framework, focusing on people and communities to provide coordinated health care that meets specific needs and promotes universal health coverage (Khatri et al., 2023). In addition, it empowers and engages patients in self-care, moving away from the passive receipt of health care services typically provided by hospitals. This community-based MIH care model emphasizes improving patient outcomes and optimizing resource utilization, transitioning from a treatment-centric and volume-based health care system to a human-centric and outcome-based delivery model, specifically referred to as value-based health care (VBHC) (Porter & Lee, 2016).

Consistent with the opening commentary (Balasubramanian et al., 2026), MIH demonstrates how ageing care innovation depends on linking workforce redesign, value-based service models, and place-based care delivery into a coherent system.

## Research Context

The empirical setting of this study is a 1.1.-31.12.2024 MIH project in North Ostrobothnia, Finland, focusing on acute care for the elderly. The Wellbeing Services of North Ostrobothnia is one of the 21 Wellbeing Services Counties in Finland, serving a population of ~416,000 residents. Wellbeing service counties in Finland differ from administrative units and include multiple administrative areas. Since January 1, 2023, Finland’s wellbeing services counties have assumed responsibility for health care, social welfare, and rescue services within their respective regions. Predominantly funded by the central government through taxation (Keskimaki et al., 2019), the operating structures and practices are created to generate people-oriented service entities. The reform seeks to reinforce publicly funded service provision by promoting equitable access, enhancing quality, and mitigating regional disparities. It also addresses systemic pressures such as demographic aging and workforce sustainability (Ministry of Social Affairs and Health, 2025).

Finland’s health care payment structure reflects a Nordic model, emphasizing universal access, equity, and cost-sharing. By using fixed client fees and capped annual payments (medical transportation is not part of the capped services), health care and social services demonstrate a strong commitment to solidarity and social justice (Ramsdal & Bjørkquist, 2020). Standardized fees through a payment ceiling ensure that health care remains accessible without overburdening public finances. Financially, the system aims for affordability for individuals (https://stm.fi/en/client-fees).

The MIH was implemented as a collaborative approach between emergency medical services (EMS) and elderly care services in the wellbeing county of North Ostrobothnia. Operationally, the MIH unit was positioned between traditional emergency care and home care, and its specific objective was to respond to unplanned and urgent care needs, mainly in patients’ homes. The MIH unit was in daily use in the urban area from 10 a.m. to 10 p.m., where it operated alongside emergency medical services units, five hospital-at-home units, and multiple home care units. The unit’s staff consisted of two nurses, a paramedic, and a geriatric nurse, who worked together to assess and treat older adults in their homes and residential settings, and, when necessary, to coordinate further care, such as referral to hospital-at-home services.

**FIGURE 1:**
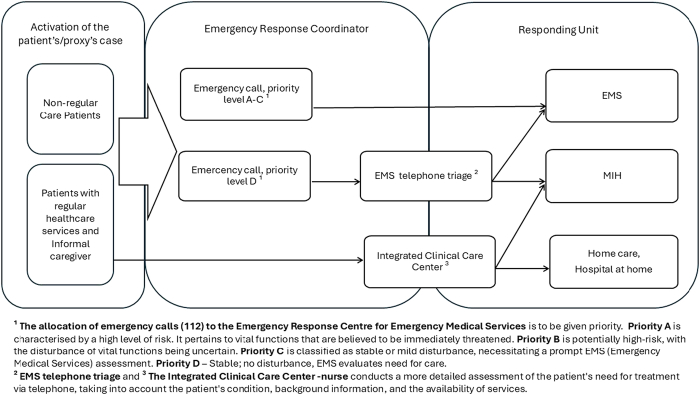
Process for activating MIH service.

Figure 1 illustrates the task activation process for the MIH unit. The main initial incident concerned the group of elderly patients, regardless of whether they were still receiving regular services. The MIH was only dispatched to patients following a professional assessment of care needs, either through EMS telephone triage or the Integrated Clinical Care Center. In certain cases, the emergency response center was able to alert the MIH unit directly to health care facilities.

The MIH unit was equipped with more advanced equipment than standard EMS units, such as comprehensive point-of-care tests (CRP, electrolytes, hemoglobin) and the ability to take venous blood samples. These resources enabled more effective assessment of patients, initiation of treatments such as antibiotic therapy, and coordination of follow-up care. If necessary, physicians could also be consulted for treatment guidelines and care planning.

In Finland, health care professionals (HCPs) are categorized as either licensed practitioners or those with a protected occupational title (The National Supervisory Authority for Welfare and Health, 2025). HCPs play a central role in providing MIH care, ensuring quality, and adopting new models and digital tools to improve efficiency and accessibility; however, their roles are often overlooked in VBHC, which emphasizes patient outcomes (Hovlin et al., 2022; Van Engen et al., 2022). This study aims to link patient-centric outcomes to the value creation process of service providers and to investigate integrating VBHC into the MIH for advancing human-centric elderly health services.

This study investigates resource reconfiguration from the perspective of HCPs in MIH, reflecting on their redefined tasks, role changes, required skills, and interactions with patients. The study employs a transdisciplinary approach, using value creation to understand VBHC in MIH. The research question is:

“*How does the MIH model create, deliver, and capture value-based health care for patients and service providers?”*

## Conceptual Framework

### Value-Based Health Care

The increasing aged population, coupled with reduced health care budgets and concerns regarding overdiagnosis and overtreatment, has sparked discussions on VBHC (Porter, 2008). In his earlier study on value in health care, Porter focused on the cost calculations associated with care provision (Porter, 2010); however, he did not sufficiently address the perceptions of health service relationships and patient experiences (van Staalduinen et al., 2023). Subsequently, Porter advanced the concept of VBHC, which emphasizes prioritizing patient needs, outcomes, and overall experience of the services provided to patients, and cost-effectiveness (Porter & Lee, 2016). The reimbursement model in health care is based on the experiences and outcomes derived from the entire patient-centric cycle (Porter, 2008). This paradigm shift signifies a transition from a volume- and cost-based approach to a more human-centric and value-based model through optimized health resources.

Patient-centricity has emerged as a fundamental principle in the delivery of health care services within a value-based framework. Most studies grounded in service logic prioritize the needs and experiences of patients. However, improvements in patient outcomes and experiences do not always result in increased efficiency and cost savings for health care providers, nor do they ensure a positive experience for HCPs. Conversely, enhancing the efficiency of health care service processes may not necessarily lead to improved patient experiences. This study articulates value through a value-based approach that addresses both the patient and the service provider, emphasizing the reciprocal nature of their relationship. Consequently, the value propositions of this paper include adopting the VBHC approach in the MIH to enhance patient experience while simultaneously increasing care efficiency, cost-effectiveness, and HCPs’ experience for service providers within the care pathway.

### Value Creation and Resource Configurations

Value creation, delivery, and capture describe the process by which organizations create value for customers while capturing value within the organization to ensure profitability (Minerbo & Brito, 2022; Teece & Linden, 2017). In addition, effective delivery of value enhances customer experience and increases customer retention. Value creation involves the design and development of products and services that address customer needs or solve their problems (Andreassen et al., 2016). Value delivery is the process of making sure that the value created is effectively and efficiently provided to the customer, which will improve the overall experience of the product or services (de Ruyter et al., 1997). Value capture pertains to an organization’s capability to generate revenue from the value it has created and delivered as outcomes.

The sources and types of value are essential dimensions of value creation. The sources of value, which include products, information, interactions, environments, and ownership, comprise the fundamental elements, activities, or resources that enhance value for organizations and stakeholders while influencing the value perceived by customers. Value creation from these sources results in cost, functional, and experiential types of value (Lindman et al., 2016). The value creation derived from value sources pertains to the content, structure, and governance activities of an organization, which are connected to its resource configuration and dynamic capabilities (Amit & Han, 2017; Kokshagina, 2021; van Staalduinen et al., 2023). Value configurations refer to the design process through which public service users create, deliver, and capture value (Eriksson et al., 2020; Trischler et al., 2017). The sources of value can be realized by identifying, matching, and bridging newly identified needs with existing resources (Teece, 2018; Teece & Linden, 2017). The resource configuration approach to value creation emphasizes the process through which existing resources can be leveraged to explore new opportunities and create value for organizations (Amit & Han, 2017).

## Methods

### Data Collection

This study examines VBHC in MIH and how service providers have reconfigured resources to enhance value for elderly acute care. A qualitative methodology is used for an in-depth analysis (Elo & Kyngäs, 2008; Hsieh & Shannon, 2005). A purposeful sampling strategy was used in this study. A doctoral researcher involved in the design and development of the MIH unit contacted all HCPs working in the unit (*N* = 23). One did not respond, and another had a scheduling conflict. Ultimately, 21 HCPs participated in the interviews. All participants had experience either in the MIH unit, the EMS telephone triage, or the Integrated Clinical Care Center.

To protect participant anonymity, only aggregated demographic data are reported in Table 1, including age, gender, specialization in nursing, and experience in both MIH and general health care work. Semistructured, face-to-face interviews (Creswell & Poth, 2016) were conducted in Finnish, tape-recorded, and transcribed by the doctoral researcher.

**TABLE 1:** Demographic data of the participants

Number of participants	21 HCPs
Gender distribution	14 females, 7 males
Average age	41.3 years
Age range	28–57 years
Educational background	9 nurses and 12 paramedics
Average duration of MIH role	12 months
MIH experience range	5–36 months
Average health care experience	16.2 years
Health care experience range	7–35 years
Average interview duration	54:33 min
Interview duration range	37:44 – 69:55 min

### Data Analysis

Thematic analysis was used to examine the data collected from the interviews (Fereday & Muir-Cochrane, 2006; Kallio et al., 2016). This method involves systematic identification, analysis, and reporting of patterns (themes) within the data (Braun et al., 2019). The process began with familiarization, during which the transcripts were reviewed multiple times to gain an understanding of the MIH care pathway and its activities before the coding process commenced. Next, codes were assigned and labeled to capture the meanings relevant to the research question. Subsequently, these codes are grouped into potential themes to identify patterns and broader concepts emerging from the coded data. The themes were then refined by merging, splitting, or discarding to ensure accurate representation of the data (Terry et al., 2017). Finally, the themes were organized in a sequence that reflects the stages of venture capture, delivery, and capture to present our research findings.

Themes in value creation included human resources, dynamic ED and social network resources, and the standardization and legitimacy for effective and efficient MIH implementation. Themes in value delivery included the channels through which MIH services reach patients, as well as areas for potential service improvement. Themes in value capture included the value perceived by patients, HCPs, and service providers of MIH. The coding structure is presented in Table 2 and more information on rigor and reflexivity is available in available in Appendix X, Supplemental Digital Content 1, http://links.lww.com/HCMR/A206.

### Ethical Considerations

This study adheres to the ethical principles outlined in the Helsinki Declaration. Informed consent was obtained from all participants before the interviews, ensuring their voluntary participation. Finnish law did not require approval from a research ethics committee, as the study did not involve clinical trials, minors, or any potential physical or psychological harm (Medical Research Act No. 488/1999).

## Results

*Value creation* for MIH services is achieved through three categories: human resources, dynamic ED and social network resources, and standardization and legitimacy of the MIH care pathway. Human resources include HCPs´ roles and work change, multidisciplinary nurse pair work, and necessary training. The dynamic ED and social network focus on mobility and technology, while standardization and legitimacy involve patient groups, MIH treatment guidelines, and the scope of home-delivered services.

### Human Resources

Change in HCPs´ care work for MIH is highlighted in the HCPs’ interviews. Remote patient evaluation and consultation via phone calls is a new responsibility for paramedic nurses. One nurse remarked, “*In ED, I typically respond to the scene, administer first aid, and transport patients to the hospital without hesitation. However, in MIH, it is not always advisable to use emergency services for patients, I should make judgment, provide the needed care, and if necessary, consult with doctors and recommend that patients visit health centers the following day* *”* (Participant 08). Essential soft skills include active listening, empathy, compassion, and understanding, which help build trust and identify health issues based on patients’ descriptions. Nurses must also make independent decisions considering patients’ health and social situations to find the best health solutions. *“* *We should trust what the patient says and make them feel comfortable, not worried, and calm down the situation, meanwhile judging from their words* *”* (Participant 04).

Multidisciplinary nursing pair work improves health service delivery in patients’ homes. Paramedic nurses identify urgent situations and provide first aid for the elderly, while geriatric nurses offer extensive care and possess a deeper understanding of patients’ needs and social support networks. Shared responsibilities enhance confidence and reduce the burden of independent decision-making, while knowledge exchange improves health decision-making for patient care. *“It is some kind of fast and hectic work, a paramedic can make right and fast judgement for emergency case”* (Participant 02). “*MIH is not as straightforward as ‘cut the wood, pile the wood, and use the wood,’ and transport patients to the ED. It involves the use of infusion and pain pump medications that are not usually included in paramedic work, as well as the follow-up service, who to call, and network in the geriatric network*” (Participant 07).

Training is crucial for HCPs in MIH due to evolving job roles and contemporary nursing practices that emphasize soft skills and comprehensive patient care, and the big picture of hospital and surrounding network support. “*There are always new things in the care of the elderly, and everything should be updated regularly*” (Participant 20). Additional training is required for conducting medical tests, using devices, and administering medications at home. “*However, it would be beneficial to leverage our expertise, as we often receive inquiries regarding pain pumps and related issues. It is crucial for us to have some guidance on how to properly secure them, among other considerations”* (Participant 02). MIH also needs proficiency in health care data management, highlighting the need for improved digital competencies. *“* *We need to use more systems to retrieve patient data, EHR, emergency care system, social care system and other medical devices*” (Participant 03).

### Dynamic ED and Social Care Network Resources

HCPs noted that dynamic ED network resources for MIH are vital for effective service delivery. Physicians, with their knowledge and understanding of the physical condition of elders and their familiarity with various basic diseases, are needed to treat elders at home. Timely consultations with geriatric and ED physicians, combined with a clear understanding of resource availability, would significantly enhance MIH practices.

Situational awareness of a patient’s living environment enables MIH providers to identify potential health issues. Coordination with home care services enhances understanding of a patient’s health, allowing for personalized care and informed decisions: “*…can more accurately assess the home environment and perhaps the modifications needed there and what the patient needs to be able to cope at home*” (Participant 11).

Collaboration with social care workers and caregivers effectively addresses these issues, supporting elderly patients. While MIH manages acute diseases, a well-organized follow-up process is crucial for optimal care. Thus, integrating social and health services among providers, home care, and caregivers should be prioritized in MIH services.

“*KOTAS (a situation center for older adults and HCPs dealing with urgent and non-urgent situations—excluding emergencies) possesses extensive knowledge about available resources and their potential applications. In contrast, EMS telephone care assessment specifically handles non-urgent medical emergency telephone calls from the emergency dispatch center and communicates with the ED regarding appropriate emergency procedures, the types of examinations that should be conducted, and the limitations of the service. Home care plays a crucial role, as it understands the needs of elderly individuals, and their initial evaluation significantly influences our assessment of the services, and the care required. Together with social and crisis services, we can collaboratively find a way to help elderly patients live independently at home by considering their current health conditions*” (Participant 11).

Technology enhances the mobility of MIH HCPs outside hospitals. Connecting various HISs, EHRs, and social care systems for patient information is essential for supporting the MIH network among EDs, hospitals, and social care services, enabling efficient patient information retrieval for decision-making. “*…well streamline, yes I find that access to patient information systems streamline so much that I can see the patient’s permission to his background information, diseases, medications and the fact that what kind of ability to function is, whether there is what kind of medical history, so it easily affects the decisions or the fact that which service I direct him to, so the background information is*” (Participant 11).

Kela provides services, such as taxi and order center assistance, to help elderly individuals travel from home to hospitals, alongside ambulance services based on health conditions. “*Do they know that you can go to the emergency room with a Kela taxi, they have not known, but they usually think that you just go to 112 and that’s how you get there. And people still think that an ambulance will get you to the operating theatre past the queues*” (Participant 09).

### Standardizing the MIH Care Pathway

The results show that target patient groups for MIH are not clearly defined. HCPs noted risks for elderly individuals who are immobile, have dementia, or deteriorating health, including those with alcoholism and mental health disorders, when managing acute illnesses at home. Challenges also exist in addressing the needs of patients who refuse mandated care. “*Monitoring patients after MIH especially for those who live along with alcoholism or dementia is challenging, because being at home should be self-activated, which is different from treatment at patient wards as always HCPs follow and with them*” (Participant 14).

The HCPs recognized the need to standardize the MIH care pathway, particularly regarding protocols and treatment. Clear distinctions among health services—like emergency services, hospital-at-home programs, home care, and social care—are essential for optimizing resource use and maximizing spillover effects. “*It is a lot of thought about which would be the right unit to come, as it is not always the same. Just think about whether it is the home care visit, MIH, emergency care, or home hospital visit. It also depends on what resources are available, but which is the most sensible to deliver there, in that sense*” (Participant 04).

*Value delivery* for MIH involves three categories: information delivery, service delivery, and continuous improvement. Information delivery focuses on outreach, changes in emergency service attitudes, and perceptions of home treatment. Service delivery emphasizes patient and health care provider experiences for service enhancement. Continuous improvement addresses current challenges and future opportunities for MIH as an alternative emergency care pathway for the elderly.

### Information Delivery

The HCPs have reported misconceptions surrounding emergency services and the health care provided by ambulances. Patients may mistakenly believe that an ED physician is always available in an ambulance, and it is the easiest way to receive quality care than other health services. “*Sometimes I have heard that elderly people think that every ambulance has a doctor, they have such a wrong idea. so that’s probably how they think that ambulances are treated by doctors. so that’s why they ‘order an ambulance’*” (Participant 11).

Another aspect highlighted by the HCPs is the necessity for patients to self-activate and manage acute conditions after receiving treatment at home following MIH interventions. This approach differs from the care provided in general wards, where HCPs consistently monitor and support patients. “*Elderly people who are extremely frail pose the highest risk of receiving home care, as they are incapable of self-care. Nobody can take this type of risk to treat them at the patient’s home, e.g., Alzheimer (some parts of patients can, but some cannot)*” (Participant 09).

The HCPs have brainstormed strategies to effectively reach target groups and promote MIH and its services in a more efficient and accurate manner. *“* *To develop an impactful solution, we must consider both the quantity (of patients) and the resources required to reach our target audience*” (Participant 18). *“* *The more this is done, the more people become aware of it and the older person knows that it is possible to get services at home and everything. and maybe the change in thinking that not everything requires an emergency room visit or a hospital stay* *”* (Participant 11).

### Service Delivery

Service delivery emphasizes both patients´ and HCPs´ experiences and satisfaction. The overall experience associated with MIH is highly valued due to its clinical support, psychological support, and the reduced burden on caregivers, who are often elderly and inconvenient in accompanying patients to the ED. *“* *They (elderly patients) are appreciated and satisfied, and they do not complain about not being able to hear or about not receiving treatment for all their concerns and issues in the emergency department despite the long wait times*” (Participant 03).

The overall experiences reported by HCPs are also positive due to the dynamic support provided by multidisciplinary nursing teams, the availability of network resources, and a work environment that is more flexible, dynamic, and collaborative than before. However, challenges related to HCP service include independent health decision-making and judgment based on patient input. “*You don’t stay alone with your decisions, even though they are not necessarily big ones, but you could discuss them more openly, you might develop ability of decision-making better*” (Participant 08).

### Continuous Improvement

The challenges associated with MIH include an increased workload, a lack of medical devices, and the absence of home-use tests. Suggestions have been made to implement mobile imaging medical vehicles to support service delivery. “*I think that from the perspective of emergency care, there are many people who could be treated at home if the equipment were available. Patients are transported to the ED because we can’t get bedside analytics like hemoglobin and C-reactive protein (CRP), and even if we do, there’s no possibility to start antibiotics or take a urine sample*” (Participant 16).

There is potential for the misuse of MIH services, particularly in ordering nonemergency health services that could be treated at nearby health centers. Future emphasis should be placed on resource allocation and funding to further develop multiagency field commanding systems to monitor, assist, and follow the process.

*Value capture* for MIH services has two dimensions: patient-perceived value includes human-centric and personalized care, convenience, and responsiveness to their care needs, along with social and psychological support. The service provider-perceived value, as recognized by HCPs, includes optimized ED patient pathways, which result in reduced emergency visits and hospitalizations, and the collaborative effects of the ED and social care service network.

### Patient-Perceived Value

Human-centric and personalized care that takes into account the social and living environments of elderly patients is essential. This approach benefits those who struggle with transportation to the ED and often require additional caregivers. Responsiveness involves providing comprehensive health care that addresses multiple health concerns rather than focusing on a single issue. This model allows for thorough checkups and attentive listening to patients’ stories, which offers emotional and psychological support and reduces the feelings of uncertainty associated with waiting in the ED and hospitals. In addition, avoiding unnecessary visits to the ED minimizes the risk of cross-infection. “*of course it avoids the hospital visit, avoids with good luck a long trip to the provinces, an expensive stay in the ward and is able to temporarily increase home care and start antibiotics*” (Participant 21). “*The satisfaction is huge. Chronic patients can now receive antibiotic treatment and blood tests at home. Patients should have a spouse accompany them on hospital visits, as they may be elderly and lack stamina to do so themselves*” (Participant 13).

### Health Service Provider-Perceived Value

The implementation of an optimized care pathway enhances efficiency by allowing the ED to concentrate solely on emergency cases. “*It reduces the burden on the emergency room and healthcare system, where elderly people lie there for hours and hours, taking up essential staff resources*” (Participant 13). *“* *This cost-effective solution reduces the burden on urgent care services and emergency departments*” (Participant 09).

This approach is expected to decrease both ED utilization and waiting times, thereby positively impacting patient wards. MIH, as a supplementary service within the elder care pathway, effectively leverages emergency human resources and fosters high levels of satisfaction among HCPs. “*Managing health issues at home is often easier than frequent emergency room visits, saving time and money while allowing for more efficient resource use*” (Participant 04).

The reduction of avoidable ED visits, coupled with improved collaboration within the health network, may enhance cost-effectiveness across the entire continuum of elder patient care. *“* *If it starts with a home care call for health services, and then home care gets involved, it usually doesn’t stop there—it keeps calling other services, and then there are return calls, back and forth. So, those chains of care can get really long. But if you have MIH in the mix, MIH moves things forward, so the chain becomes much shorter and much more efficient* *”* (Participant 15).

## Discussion

### MIH Service as Intentional Value Creation

The study addresses a critical research gap in understanding the transition from cost-based care to VBHC, conceptualizing MIH care services as an intentional process of value creation, aimed at delivering human-centric health emergency services in community-based settings. Table 3 Appendix X, Supplemental Digital Content 1, http://links.lww.com/HCMR/A206 outlines the strategy for value creation, delivery, and capture in managing MIH services by identifying, matching, and bridging needs through existing resources to enable human-centric elderly care.

The study highlights the role of MIH HCPs and the combination of primary emergency care with community-based resources as sources of value for patient-centered care for the elderly at home. The study focuses on task-shifting (Das et al., 2024), the utilization of multiprofessional nursing skills (Duffield et al., 2010), and service enhancement through technology and health data platforms.

The skills needed for MIH HCPs who work with elderly patients include soft skills such as active listening and empathy, as well as the ability to make effective treatment and care decisions through telephone-based patient evaluations. Soft skills are crucial not only for leading health care organizations (Enard et al., 2025) but also for providing quality patient care. The value creation is further enhanced through multidisciplinary nursing collaboration and the complementarity of diverse care knowledge and capabilities in decision-making at patients’ homes. This MIH project aligns with the WHO Europe and Care Workforce Framework for Action 2023–2030, optimizing performance and efficiency among the limited health workforce by reconfiguring services and redefining teams to maximize human resources and ensure value-added actions (Azzopardi-Muscat et al., 2023; Organization, W. H., 2023).

Given the complex health and social challenges faced by the aging population, this study emphasizes the importance of developing a dynamic network of ED resources and fostering close collaboration with social care services to enhance the capacity and capability of the MIH service delivery. The situational awareness of elderly patients’ living environments and social issues represents a significant value of MIH, distinguishing the MIH model from the current ED service model provided in hospitals. However, this can only be achieved through strong connections with emergency care, paramedic health services, social care, home care, and other relevant stakeholders. This research supports the effectiveness of MIH in addressing the health-related social needs of the elderly (Miller et al., 2023) and confirms its safety in managing undifferentiated acute complaints among older patients (O’Connor et al., 2023).

The value of MIH for elderly patients in acute care has been captured by both patients and HCPs, who express satisfaction with this new model. However, continuous improvement should be prioritized in standardizing the MIH care pathway. This includes providing clearer care instructions, a more responsive attitude toward MIH services (Manias & Street, 2000), promoting self-activation and self-care, ensuring the availability of practical supportive medical testing equipment, and managing the excessive workload of HCPs.

### Integrating Value-Based MIH Into Existing Care Systems

Human centricity and outcome-based health care are essential components of VBHC and can be achieved through intentional value creation in public health services. The aspect of human centricity achieved from the MIH services includes convenient treatment at the patient’s home, timely responsiveness, comprehensive treatment, situational awareness of the patient’s living environment and social conditions, particularly psychological support that emphasizes being listened to, respected, and cared for.

The VBHC reimbursement model emphasizes the entire patient cycle, requiring integrated health care services (Porter 2008). MIH offers a scalable framework that connects governance, services, professionals, and patients in a cohesive and cost-effective system. This aligns with the IPCHS framework in promoting people and community-based sustainable health care and increasing equitable access to health care through the effective utilization of the care workforce (Abrashkin et al., 2016).

At the governance level, MIH can be integrated into Finland’s centralized, publicly funded health care and social welfare system across hospital, regional, and national levels. The implementation of MIH also requires changes in clinical practices, the development of new patient care protocols, and the legitimation and normalization of MIH within existing care structures. Integration can be further strengthened through investment in ICT infrastructure and national data platforms that enable access to patient data and support clinical decision-making within a regulatory framework, as suggested by the findings.

Integration at the service level ensures that patients receive timely interventions without unnecessary hospitalizations, aligning with value-based care principles. MIH can address acute conditions among the elderly at home, helping to reduce the burden on ED (Abrashkin et al., 2016). It bridges gaps between EMS, primary care, and social services, enabling seamless transitions from acute treatment to follow-up care, reducing duplication and enhancing continuity. MIH should be organized as an integrated and human-centric health care service rather than a separate unit and service. It demands strong commitment from hospital leadership to restructure resources and workflows.

At the HCP level, MIH serves as a bridge between ED physicians and social and community care workers. It enables task-shifting to social workers and less specialized professionals (Aithal & Aithal, 2017), who can improve elderly living conditions and provide essential support for daily activities, addressing acute health issues rooted in social and environmental factors. Task-shifting enhances access to care by optimizing the use of available human resources. MIH requires interdisciplinary collaboration among paramedics, nurses, physicians, care coordinators, and social workers. It redefines professional roles to deliver acute care in home settings, particularly for community paramedics operating beyond traditional emergency care. Effective integration at the HCP level demands shared protocols, targeted training, and functional communication systems to ensure coordinated decision-making and accountability.

At its core, MIH is patient-centered. It delivers care directly to the patient at home, respects individual preferences, and reduces barriers to access. For elderly patients, MIH can prevent adverse effects and inconvenience associated with hospital stays, while enhancing satisfaction through personalized and home-based care. The findings also indicate that, to ensure the appropriate use of MIH and to prevent excessive or inappropriate service requests, which may lead to provider burnout and system overload, it is essential to provide accurate information about the scope and limitations of this new model of emergency care. Efforts should be made to build trust in home-based treatment and to promote patient self-activation and self-care.

### Practical Implications

This study employs a qualitative research method to examine value creation and delivery within MIH and its perceived value for acute elderly ED care. MIH in Finland serves as a case study, demonstrating that health service providers should integrate MIH services at multiple levels to support a successful transition. The focus lies in developing HCPs’ skills and competencies, utilizing multidisciplinary nursing, and enhancing coordination and collaboration across social and health care services within broader network settings. Attention should also be given to the gradual standardization of services to support value creation within MIH.

The study emphasizes the importance of collaboration, information exchange, and experiential learning as mechanisms for the continuous improvement of value delivery. The MIH service is staffed by experienced paramedics and nurses who work independently in patients’ homes. Finland’s advanced digital infrastructure enables seamless connectivity between health care systems, both in hospitals and remote settings (Hyppönen et al., 2019). To support the development of MIH and broader health services, it is important to develop integrated information systems that minimize fragmented IT infrastructures and data silos. This requires not only technical development but also the establishment of clear regulations for patient data sharing to enhance care coordination across the entire patient pathway (Koivuluoma et al., 2022).

Financially, MIH offers a cost-effective alternative to traditional acute care pathways. Finland’s health care system is grounded in the Nordic welfare model, wherein health is regarded as a shared societal responsibility. MIH aligns with this principle by proactively addressing acute needs within the community among the elderly, thereby reducing pressure on the health system and promoting equity. It aims to optimize resource utilization while maintaining high-quality care through publicly funded services and fixed client fees. This model offers an alternative to more individualistic health care arrangements, demonstrating how collective investment in preventive and community-based care can lead to improved outcomes and reduced costs. For policymakers and practitioners in insurance-driven systems, the Finnish MIH approach presents an example case for redesigning acute care delivery, encouraging a shift from reactive, high-cost interventions to integrated, patient-centered models that prioritize accessibility, affordability, continuity, and sustainability.

This study presents a single-country case analysis of MIH in Finland, and its findings may not be generalizable to other health care systems. Future studies could compare publicly funded MIH models with those operating in private or insurance-driven systems to explore alternatives in organization and delivery. HCPs were selected to ensure depth and consistency in examining MIH implementation and perceived value. While their direct involvement offers valuable operational insights, the sample’s homogeneity may limit the diversity of perspectives and reduce the transferability of findings to other professional groups or settings. Future research should aim to include a more diverse sample to capture a broader range of views, for example, patients, and enhance the generalizability of insights across different health care systems and stakeholder groups. This research is qualitative in nature and explores the value creation process through which value is created, delivered, and perceived. A future registry-based study is currently being planned to evaluate the effectiveness of MIH and to support its operational development.

## Supplementary Material
