# Supplementary material for: Value-based health care in mobile integrated health for acute elderly care: A qualitative study of health care professionals in Finland
Source: Health Care Manage Rev. 2026 Jul 2;51(3 Suppl):S14–23. doi: 10.1097/HMR.0000000000000483 (PMC13326928; doi:10.1097/HMR.0000000000000483)
Supplement: Supplementary file 1 [file hmr-51-s14-s001.docx]

Table 2: Structure of data analysis

| **Theoretical lens** | **Main category** | **Generic category** | **Subcategory (Code)** | **Code Count** |
| --- | --- | --- | --- | --- |
| **Value Creation** | Human Resources | Changes in care work | Remote patient evaluation and consultation | 17 |
|  |  |  | Soft skills | 15 |
|  |  |  | Independent decision making and situation awareness | 20 |
|  |  | Multidisciplinary nurse pair work | Knowledge complementarity | 19 |
|  |  |  | Knowledge and responsibility sharing | 19 |
|  |  | Training | MIH care-related training | 11 |
|  |  |  | Technological training | 6 |
|  | Dynamic ED and social network resources | Network and mobility | ED and ED physicians | 13 |
|  |  |  | Social care service and social workers | 15 |
|  |  |  | Home care services and home care workers | 18 |
|  |  |  | Geriatric department and doctors | 15 |
|  |  |  | Hospital-at-home services | 21 |
|  |  |  | Caregivers | 11 |
|  |  |  | Transportation companies, e.g., *Kela supported taxi | 4 |
|  |  | Technology | Computer, phone and remote consultation | 18 |
|  |  |  | Patient portals e.g., Hospital Information System (HIS)/ Electronic Health Record (EHR), social care and multi-agency field commanding system | 20 |
|  |  |  | Phone call and text message in provider-to-provider communication, and provider-to-patient communication | 17 |
|  | Standardization and legitimacy | Patient groups | Elderly patients who can manage their care independently or with a caregiver | 16 |
|  |  |  | Elderly patients with no complication of health issues, mental issues or dementia | 21 |
|  |  |  | Elderly patients who cannot move or need regularly chronic or palliative care | 18 |
|  |  | MIH treatment guidelines | Conditions under which patients should be treated at homes | 12 |
|  |  |  | Phone call evaluation protocol | 11 |
|  |  |  | Protocols for treatment and care | 9 |
|  |  | Scope of home-delivered services | The care boundaries between ED, MIH, Hospital-at-home and home care | 9 |
| **Value Delivery** | Information delivery | Target group reach | Service marketing within hospitals | 11 |
|  |  |  | 116117 care hotlines | 2 |
|  |  |  | Social media, like Tiktok | 2 |
|  |  |  | Medical magazines (target for elders and elder care) | 5 |
|  |  | Attitude change of emergency services | Misunderstanding the use of ambulance | 6 |
|  |  |  | Differences between ambulance and MIH service | 4 |
|  |  |  | Fear associated with paramedic and nurse home visit | 2 |
|  |  | Attitude change of being treated at home | Trust of MIH service | 9 |
|  |  |  | Self-activated, which is different from treatment at patient wards as always HCPs follow and with them | 7 |
|  |  |  | Waiting time of two hours | 4 |
|  | Service delivery | Patient experience and satisfaction | Personalized clinical support | 12 |
|  |  |  | Psychological support | 10 |
|  |  |  | Reduced the burden of caregivers | 8 |
|  |  | HCP experience and job satisfaction | Work-well being | 8 |
|  |  |  | Meaningfulness of work | 13 |
|  |  |  | Flexibility, dynamic and collaborative way of working | 12 |
|  | Continuous Improvement | Challenges | MIH service not known | 5 |
|  |  |  | The misuse of MIH service | 7 |
|  |  |  | Increased workload of MIH | 12 |
|  |  |  | Lack of mobility for medical devices | 9 |
|  |  |  | Lack of mobility for point-of-care testing | 8 |
|  |  | Future | Learning from the pilot project and experience | 4 |
|  |  |  | More funding and resource allocation | 11 |
|  |  |  | System and management support | 4 |
| **Value Capture** | Patient perceived value | Human-centric and personalized care | Home living environment | 14 |
|  |  |  | Social factors consideration | 12 |
|  |  |  | Follow-up care arrangement, e.g., hospital at home | 13 |
|  |  |  | Connecting hospital network with social worker support | 6 |
|  |  | Convenience | Do not need to transport to ED | 19 |
|  |  |  | Do not need to wait at ED | 17 |
|  |  |  | Do not need caregiver accompanies at ED | 11 |
|  |  | Responsiveness | More comprehensive check-ups at home | 14 |
|  |  |  | More time spent on treatment and listening to their stories | 9 |
|  |  |  | Shorter time of receiving treatment and care | 10 |
|  |  | Psychological support | Avoid the fear feelings of being in ED/hospitalized | 4 |
|  |  |  | Feel like being heard, cared and respected | 6 |
|  |  | Patient safety | Reduced the adverse events in ED or hospitals | 4 |
|  |  | Affordability | Cheaper than ED service | 5 |
|  | Service provider perceived value | Efficiency | Optimized care pathway | 7 |
|  |  |  | Shorten the care pathway | 6 |
|  |  |  | ED focuses the “emergency” work | 6 |
|  |  | Effectiveness | Reduced avoidable ED visits | 17 |
|  |  |  | Reduced avoidable hospitalization | 8 |
|  |  |  | Reduced avoidable queues for ED | 3 |
| *Note: Kela, the Social Insurance Institution of Finland, has a responsibility to provide basic support in life’s ups and downs to everyone covered by the Finnish social security system. (https://www.Kela.fi/Kelas-operations) | | | | |

***Rigor and Reflexivity***

The study ensured rigor and reflexivity by adhering to the qualitative trustworthiness criteria of confirmability, credibility, authenticity, reliability, and transferability (Polit & Beck, 2020). Although the doctoral researcher who collected the data and some of the participants are employed within the same department in the well-being county, the researcher, who serves as a development coordinator in the emergency medical services unit, has no influence on the interviewed HCPs who work on the frontline. The semi-structured interview questions were discussed and validated among the research team, ensuring that the standardized questions mitigate halo effects (Noor et al., 2023). The doctoral researcher further anonymized all interviewees before conducting data analysis. Data analysis was performed independently by another researcher and subsequently validated by multiple researchers to minimize familiarity bias and confirmation bias.

The purposeful sample consisted of 21 participants, out of a total of 23 HCPs working in MIH services. This high proportion suggests that the range of perspectives captured was sufficient to reach data saturation, as thematic categories remained stable across batches. Codes were also counted across transcripts until no new codes emerged, indicating that data saturation had been achieved (Hennink & Kaiser, 2022). The sample in this study was relatively homogeneous, consisting of HCPs with similar roles and experiences within one Finnish MIH context, however, homogeneity supports internal consistency, depth of thematic analysis and broader applicability (Robinson, 2014). Authenticity was preserved using descriptive quotations from interviews. Reliability was ensured by having two researchers carry out the data analysis, and the findings were verified by the whole research team. Triangulation by multidisciplinary researchers, including experts from business and health sciences, enhanced the confirmability of the findings. Thick descriptions, detailed participant characteristics, and transparent reporting enhance the potential for transferability to similar contexts.

Table 3. Strategy for value creation, delivery, and capture in managing MIH services.

|  | **Needs** | **Benefits** | **Resource configuration** | **Bridging strategy** |
| --- | --- | --- | --- | --- |
| **Patients** | Affordable care | Reduced emergency visits and readmissions | Reallocation to nurse resources | Task-shifting to nurses for elder acute care, and social workers |
|  | Comfortable and familiar environment | Increased patient satisfaction | Home environment monitoring | Use of remote monitoring and telehealth |
|  | Responsive services | Human-centric and personalized service | Support from doctors and collaboration across care settings | Integrated care pathways between hospital and home care |
|  | Accessible services | Focused target group care | Combinations of HCPs and remote services | Leveraging HCPs through multidisciplinary teams |
| **Service Providers** | Clear patient segmentation | Efficient resource use | Changes in HCP tasks and roles | Task-shifting to optimize HCP capacity |
|  | Collaboration across care pathway | Better continuity of care | Collaboration within hospital, emergency services, and home care | Cross-department coordination |
|  | Technology-enabled workflows | Improved monitoring and decision-making | Integration of digital tools and platforms | Digital support for task-shifting and remote care |
